# Median Pancreatectomy for Frantz Tumor: Management of a Splenic Artery Aneurysm by Radiological Embolization

**DOI:** 10.1155/2024/6188288

**Published:** 2024-10-23

**Authors:** Boubker Idrissi Kaitouni, Hamza Ouzzaouit, Talha Laalou, Hamza Sekkat, Mohamed Mahi, Ittimade Nassar, Omar El Aoufir, Youness Bakkali, Mouna Mhamdi Alaoui, Farid Sabbah, Mohammed Raiss, Abdelmalek Hrora

**Affiliations:** ^1^Digestive Surgical Department C, Centre Hospitalier Ibn Sina, Rabat, Morocco; ^2^Faculty of Medicine and Pharmacy, Mohammed V University, Rabat, Morocco; ^3^Central Radiology Department, Centre Hospitalier Ibn Sina, Rabat, Morocco

**Keywords:** aneurysm, case report, embolization, fistula, median pancreatectomy, splenic artery

## Abstract

Iatrogenic aneurysms of the splenic artery constitute a rare yet potentially severe complication arising from diverse medical or surgical interventions. The clinical complexity and challenging management strategies associated with these aneurysms pose significant difficulties for clinicians. This circumstance is exemplified in our case report, detailing an iatrogenic aneurysm of the splenic artery that emerged secondary to a pancreatic fistula following a median pancreatectomy performed for a Frantz tumor. The intricate clinical presentation of this case underscores the considerable management challenges posed by such iatrogenic complications.

## 1. Introduction

Aneurysms of the splenic artery are increasingly diagnosed, particularly through cross-sectional imaging modalities [1]. This vascular anomaly carries significant mortality risks, ranging from 10% to 25% in cases of rupture [2]. Traditionally, surgical intervention served as the exclusive treatment option. However, in the past decade, endovascular techniques have emerged as a viable therapeutic alternative. Boasting a technical success rate ranging from 75% to 100%, these innovative approaches exhibit markedly reduced morbidity and mortality compared to surgical interventions [3]. In this context, we present a case report featuring a young patient who, following surgical intervention for a Frantz tumor, developed a splenic artery aneurysm secondary to a pancreatic fistula postmedian pancreatectomy. Notably, successful treatment was achieved through radiological embolization.

## 2. Case Presentation

**Figure 1:**
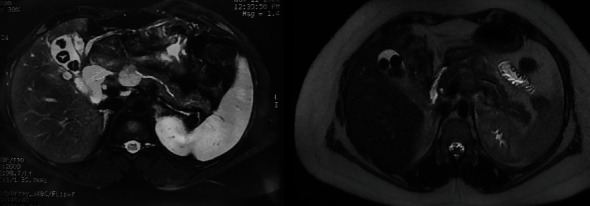
Preoperative MRI image of Frantz tumor. MRI, magnetic resonance imaging.

A 32-year-old woman, devoid of any prior medical history, was admitted to the hospital due to the presence of a cystic tumor located in the corporal-isthmic region of the pancreas. The diagnostic investigation involved pancreatic magnetic resonance imaging (MRI) and echo–endoscopy with cytopuncture, along with subsequent anatomopathological analysis. The findings indicated the presence of a solid pseudopapillary tumor of the pancreas, commonly known as a Frantz tumor measuring 25 mm × 22 mm (Figure 1). Following a multidisciplinary meeting decision, the patient was operated on by an open approach. A median pancreatectomy was performed, with connection with Roux-en-Y and closure of the proximal stump. The postoperative course was notable for the development of a pancreatic fistula on the proximal stump on the 11th day, accompanied by an elevation in lipase levels in the drainage fluid. A subsequent abdominal computer tomography (CT) scan disclosed a microaneurysm of the splenic artery measuring 5.3 mm in the long axis. Although the clinical and biological progression was favorable, the patient experienced profuse rectal discharge and an episode of hematemesis on the 25th day, necessitating readmission.

**Figure 2:**
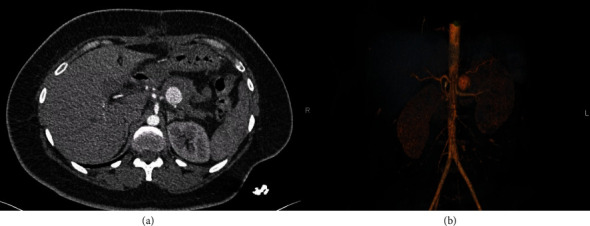
Abdominal CT angiography showing splenic artery pseudoaneurysm after central pancreatectomy. (a) Cross-sectional view. (b) 3D reconstruction. CT, computer tomography.

**Figure 3:**
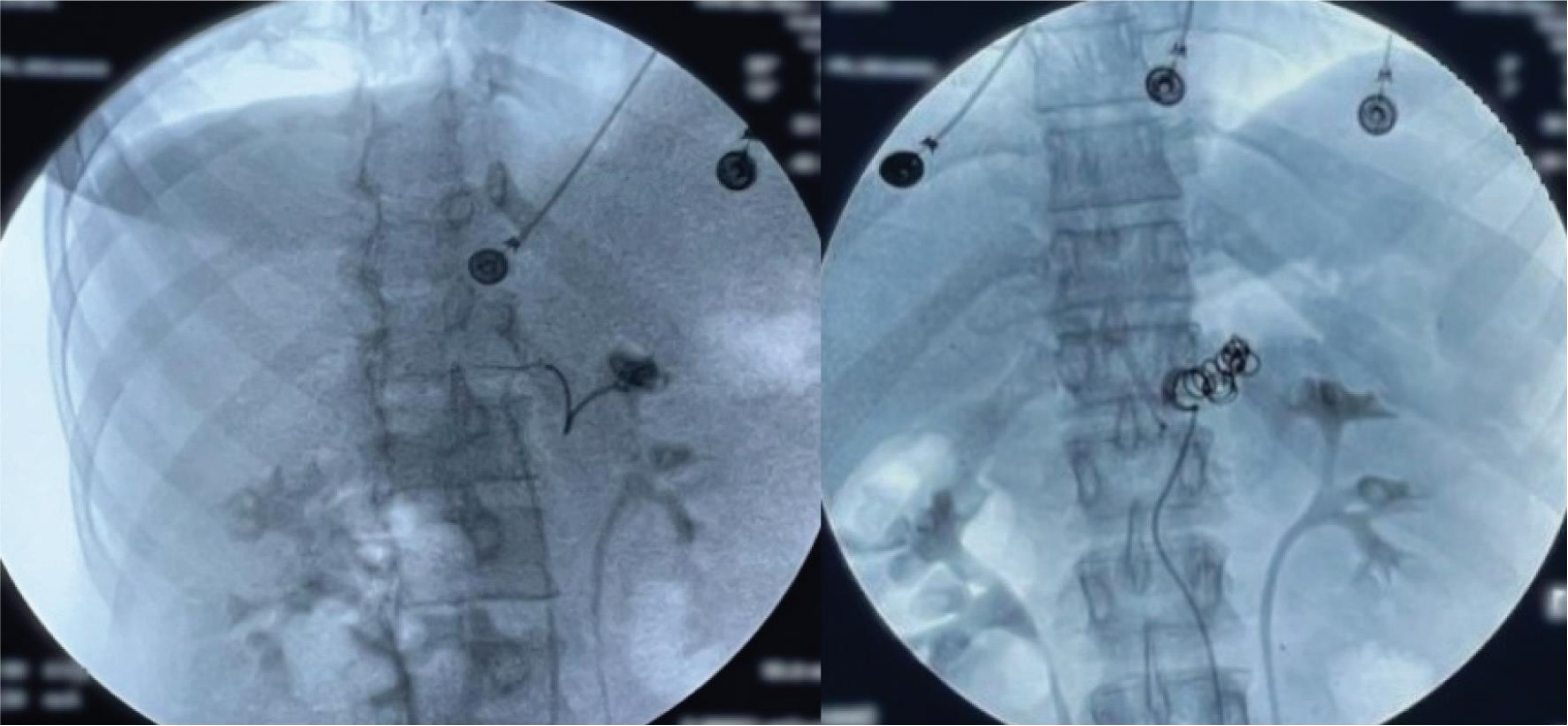
Radiological embolization of the aneurysm with placement of four endovascular coils.

The abdominal computer tomography angiography (CTA; Figure 2) unveiled a pseudoaneurysm originating from the splenic artery, measuring 23 mm × 22 mm, characterized by a narrow neck and enveloped by a parietal hematoma. The collective dimensions of the pseudoaneurysm and hematoma were measured at 44 mm × 49 mm, exhibiting close adherence to the pancreatico–jejunal anastomosis, with discernible foci of splenic infarction. In lieu of surgical intervention, and after deliberation with the interventional radiology team, the course of action involved radiological embolization through the femoral route, following the transfusion of four units of concentrated blood cells and stabilization of the patient. The procedure entailed the insertion of endovascular coils using the seldinger method (femoral approach) with a cobra probe (5F). Coiling of the artery using three coils, helical—6 mm × 10 cm (2) and 4 mm × 10 cm (1) as described in the SVS guidelines (Figure 3).

**Figure 4:**
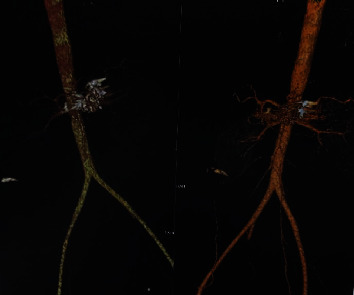
Control CT scan after radiological embolization of the aneurysm (3D reconstruction). CT, computer tomography.

**Figure 5:**
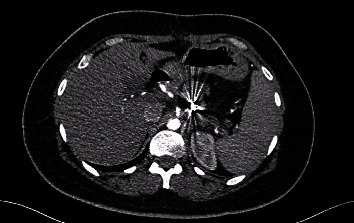
Post-embolization control CT scan at 3 months, metal coils placed. CT, computer tomography.

The subsequent follow-up was favorable, marked by the cessation of digestive bleeding and an elevation in hemoglobin levels. A follow-up CT angiography (Figure 4), conducted on the fourth day, indicated nearly complete obliteration of the pseudoaneurysm, coupled with the stabilization of foci associated with splenic infarction. The patient attended a follow-up consultation 3 months later and exhibited satisfactory recovery (Figure 5).

## 3. Discussion

Median pancreatectomy has evolved into a well-established surgical procedure, with its primary objective being the preservation of pancreatic parenchyma to maintain the multifaceted functionalities of the pancreas, particularly its endocrine functions. Additionally, even if pancreatectomy with preserving of the spleen increases operating time, this approach mitigates the postsplenectomy side effects. Indications for this surgery encompass benign and low-grade malignant lesions located in the isthmus and body of the pancreas, contingent upon sufficient surgical expertise. Despite its potential advantages, the utilization of median pancreatectomy remains infrequently carried out [4].

The most consequential complication associated with this methodology is the occurrence of a pancreatic fistula [4]. Its incidence ranges from 10% to 30% [5, 6]. This high rate of fistula is due to the creation of closed proximal slice and a distal stump anastomosing with the small intestine. Presently, mortality rates following pancreatic surgery are relatively low, ranging between 4% and 15% [7–9]. “Late” hemorrhages, occurring beyond the initial 72 postoperative hours, are predominantly associated with an anastomotic fistula in 75%– 90% of cases, often of pancreatic origin [10, 11]. These hemorrhages, which are occasionally exclusively digestive without an associated intraperitoneal component, stem from arterial or arteriolar erosion within the pancreatic slice or peripancreatic arteries. Such arterial lesions may manifest as pseudoaneurysms, characterized by a two-stage bleeding pattern—initiating with an initial, often inconspicuous bleed that halts spontaneously (“sentinel bleed”), followed by a more substantial recurrence [11].

Although aneurysms of the splenic artery are rare, they are the most prevalent among splanchnic arteries. Carmeci and Mc Clenathan [12] reported 15 cases concerning the splenic artery in a series of 31 visceral artery aneurysms. These aneurysms exhibit a higher incidence in women, occurring two to three times more frequently and are commonly identified between the third and sixth decades of life [13]. A categorical distinction is made between true and false aneurysms [14]. For the purposes of this discussion, focus will be directed towards false aneurysms, with etiology encompassing trauma [13], pancreatic pseudocysts [15], or infection of the arterial wall leading to a mycotic aneurysm [14].

Regardless of their diameter, all false aneurysms pose a noteworthy risk of rupture, emphasizing the imperative for a prompt intervention in all cases. This risk becomes significant above a diameter of 2 cm [14], especially exceeding 3 cm [16], and in false aneurysms characterized by accelerated growth [17]. Rupture of an aneurysm represents a serious complication, contributing to 10%–25% of fatalities. Hemorrhage may manifest as massive intraperitoneal bleeding or externalization in cases of rupture into adjacent structures, such as the digestive tract or pancreatic duct [10].

A literature review uncovered two additional instances of iatrogenic false aneurysms of the splenic artery. The first case, reported by Quinn et al. [18], involved percutaneous drainage of a pancreatic pseudocyst, where the drainage catheter induced splenic artery injury, culminating in a false aneurysm and subsequent hematemesis. This necessitated a laparotomy for splenectomy. The second case, documented by Michel et al. [19], detailed an iatrogenic false aneurysm of the splenic artery arising from percutaneous drainage of a retrogastric collection after cephalic duodenopancreatectomy (CPD). Surgical intervention, including splenectomy, led to successful recovery. In the presented case, a strategic decision was made to opt for radiological embolization of the splenic artery with the deployment of coils, given the proximal location of the false aneurysm. This intervention resulted in a favorable outcome, emphasizing both recovery and the preservation of the spleen.

Surgical interventions for splenic artery aneurysms encompass various techniques, typically performed through laparotomy. Carmeci and McClenathan [12] documented two cases of laparoscopic aneurysmectomy of a splenic artery. An alternative therapeutic approach involves interventional radiology, which entails the obliteration of the typically saccular aneurysmal sac by deploying coils. This intervention aims to either preserve the flow in the splenic artery or interrupt it in cases where aneurysmal rupture has occurred. Endovascular techniques have demonstrated notable efficacy, with numerous teams reporting a success rate of approximately 85% [15, 20]. Tulsyan et al. [21] documented a 100% success rate with endovascular techniques in a series of 20 splenic artery aneurysms, where 17 were treated electively and three urgently, with no recorded mortality at 1 month. Similarly, Sachdev et al. [22] reported an equal utilization of surgical and endovascular techniques in his study of 25 splenic artery aneurysms, with 85% of them being true aneurysms. The success rate for endovascular techniques in this series was 89%. Nonetheless, it is crucial to acknowledge the possibility of failures, as evidenced by Carmeci and McClenathan's [12] series where failures occurred in four out of five cases. Additionally, Kuzuya et al. [23] reported a secondary rupture 1 year after embolization.

Embolization stands out as a viable option for the treatment of splenic artery aneurysms, particularly for distal aneurysms, given its feasibility, while the use of covered stents may be constrained by arterial tortuosity [24]. However, this intervention is generally not applicable to aneurysms situated at the splenic hilum, and it is less indicated in cases of hemodynamic instability defined by the requirements for transfusion of more than two packed red blood cells [16, 23]. A variety of materials, with coils being prominent among them, are employed in the embolization process, either alone or in conjunction with other embolic agents [25], and the risk of splenic infarction following embolization is notably rare due to the robust vascular supply provided by the short gastric vessels [26]. In alignment with this approach, we opted for radiological embolization in our patient with a proximal splenic artery aneurysm, following hemodynamic stabilization and transfusion of units of concentrated blood cells.

Surveillance with CT scan 1 and 6 months after embolization is recommended [20]. In our case, we recommend CT surveillance at 3 months to monitor the evolution of the embolized aneurysm, then at 6 months for neoplastic surveillance, and then once a year in the absence of any clinical signs. Repermeabilization of splenic artery aneurysms is documented in the literature, with reported rates of 56% in Wojtaszek's series [27], 12.5% in Yamamoto's study [28], and 9% in Patel's investigation [29]. The Mayo series demonstrated a success rate of 98% for complete exclusion, while the Cleveland Clinic series reported a success rate of 97% [30]. When comparing endovascular treatment with open surgery, the former is associated with a superior quality of life and emerges as a more cost-effective strategy. Immediate advantages of endovascular treatment include local anesthesia, shorter hospital stays, and faster recovery [22].

## 4. Conclusion

Iatrogenic splenic artery aneurysm is an exceedingly rare complication arising after pancreatic surgery, posing a serious and potentially life-threatening condition necessitating urgent intervention. Endovascular treatment, particularly coiled embolization, is increasingly recognized as the standard of care, particularly when dealing with aneurysms exceeding 2 cm in diameter or those characterized as false aneurysms. Surgery should be considered if radiological embolization fails or if the patient is hemodynamically unstable, a scenario often associated with high mortality rates.

## Data Availability

The data that support the findings of patient information are available from the corresponding author upon reasonable request.
